# Withanolide D Enhances Radiosensitivity of Human Cancer Cells by Inhibiting DNA Damage Non-homologous End Joining Repair Pathway

**DOI:** 10.3389/fonc.2019.01468

**Published:** 2020-01-08

**Authors:** Jerome Lacombe, Titouan Cretignier, Laetitia Meli, E. M. Kithsiri Wijeratne, Jean-Luc Veuthey, Muriel Cuendet, A. A. Leslie Gunatilaka, Frederic Zenhausern

**Affiliations:** ^1^Center for Applied NanoBioscience and Medicine, College of Medicine Phoenix, University of Arizona, Phoenix, AZ, United States; ^2^School of Pharmaceutical Sciences, University of Geneva, Geneva, Switzerland; ^3^Southwest Center for Natural Products Research, School of Natural Resources & the Environment, College of Agriculture & Life Sciences, University of Arizona, Tucson, AZ, United States

**Keywords:** withanolide D, cancer, radiation, radiosensitizer, DNA damage repair, mitotic catastrophe

## Abstract

Along with surgery and chemotherapy, radiation therapy (RT) is an important modality in cancer treatment, and the development of radiosensitizers is a current key challenge in radiobiology to maximize RT efficiency. In this study, the radiosensitizing effect of a natural compound from the withanolide family, withanolide D (WD), was assessed. Clonogenic assays showed that a 1 h WD pretreatment (0.7 μM) before irradiation decreased the surviving fraction of several cancer cell lines. To determine the mechanisms by which WD achieved its radiosensitizing effect, we then assessed whether WD could promote radiation-induced DNA damages and inhibit double-strand breaks (DSBs) repair in SKOV3 cells. Comet and γH2AX/53BP1 foci formation assays confirmed that DSBs were higher between 1 and 24 h after 2 Gy-irradiation in WD-treated cells compared to vehicle-treated cells, suggesting that WD induced the persistence of radiation-induced DNA damages. Immunoblotting was then performed to investigate protein expression involved in DNA repair pathways. Interestingly, DNA-PKc, ATM, and their phosphorylated forms appeared to be inhibited 24 h post-irradiation in WD-treated samples. XRCC4 expression was also down-regulated while RAD51 expression did not change compared to vehicle-treated cells suggesting that only non-homologous end joining (NHEJ) pathways was inhibited by WD. Mitotic catastrophe (MC) was then investigated in SKOV3, a p53-deficient cell line, to assess the consequence of such inhibition. MC was induced after irradiation and was predominant in WD-treated samples as shown by the few numbers of cells pursuing into anaphase and the increased amount of bipolar metaphasic cells. Together, these data demonstrated that WD could be a promising radiosensitizer candidate for RT by inhibiting NHEJ pathway and promoting MC. Additional studies are required to better understand its efficiency and mechanism of action in more relevant clinical models.

## Introduction

Along with surgery and chemotherapy, radiation therapy (RT) is an important modality in cancer treatment, with about half of cancer patients receiving RT as a curative or palliative treatment. However, there are still many challenges to improve its efficiency by minimizing normal tissue toxicity while maximizing tumor control. In this perspective, an approach combining RT with radiosensitizers has been long investigated in order to allow for better dose modulation (1). With the emergence of small molecules, macromolecules, and nanomaterials, recent progresses in the field have been significant (2). However, given the severe limitations of radiosensitizers currently administered in the clinic, there is an urgent need for new targeted RT-compatible anticancer treatments.

*Withania somnifera* is a plant originated from the dry regions of Asian countries and that possesses diverse biological activities, such as anti-inflammatory, anti-stress, antioxidant, immunomodulatory, anti-angiogenic, and anticancer activities. These numerous effects are known to be due to the presence of withanolides, a class of steroidal lactones, present in the roots, and leaves of this plant (3). Thus, several studies have shown that withanolides exert their antitumor activity by inducing ROS production, cell cycle arrest, cytoskeleton destabilization, etc. (4). Interestingly, the most studied *W. somnifera*-derived compound, withaferin A (WFA), has also shown to have a radiosensitizing effect (5). Yang et al. confirmed that WFA enhanced radiation-induced apoptosis in renal and leukemia cell lines through down-regulation of the anti-apoptotic protein Bcl-2 (6, 7). This effect has also been demonstrated *in vivo*, where WFA treatment when associated with fractionated RT synergistically increased the complete response of mouse melanoma, fibrosarcoma, and Ehrlich ascites carcinoma (8, 9). Therefore, these studies suggest the great interest of withanolides as candidate cancer drugs and especially as potential radiosensitizers.

**Figure 1:**
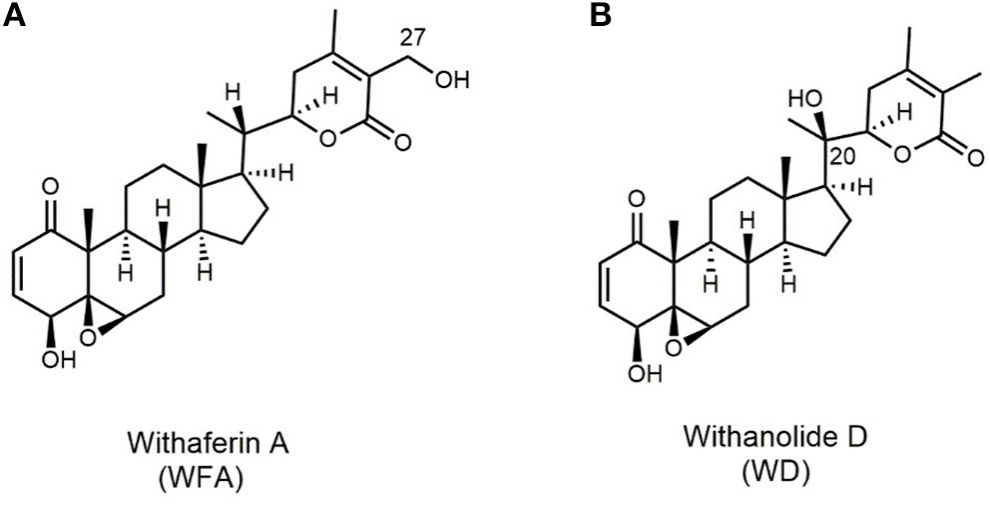
Structures of **(A)** withaferin A and **(B)** withanolide D.

In this study, the effect of withanolide D (WD) in combination to radiation on several cancer cell lines was investigated. Structurally, WD is an isomer of WFA that feature 2(3)-en-1-one, 4β-hydroxy, and 5β,6β-epoxy moieties in their A and B rings. They differ from each other only in the position of the additional hydroxy group present in their side chain (C-27 in WFA and C-20 in WD) (Figure 1).

## Materials and Methods

### Withanolides

WFA and WD were obtained from the aerial parts of aeroponically grown *W. somnifera* (10) as reported previously (11). Their purities were confirmed to be >98% by HPLC analyses (Supplementary Figures 1, 2) and proton NMR spectroscopy (Supplementary Figures 3, 4). Compounds were dissolved in DMSO to obtain 10 mM solutions.

### Cell Culture

The human ovarian SKOV3 (HTB-77), intestinal Caco-2 (HTB-37), prostate DU145 (HTB-81) and 22Rv1 (CRL-2505), lung A549 (CCL-185), and breast MCF7 (HTB-22) cancer cells, were obtained from ATCC. SKOV3 were maintained in McCoy's 5A medium, Caco-2 in Dulbecco's Modified Eagle's Medium, DU145 in Minimum Essential Medium, 22Rv1 in RPMI-1640 medium, A549 in F-12K medium, MCF7 in Minimum Essential Medium with 0.01 mg/mL insulin, all supplemented with 10% of Fetal Bovine Serum (FBS) and 1% of streptomycin/penicillin. They were incubated at 37°C in 5% CO_2_ atmosphere. Medium was changed twice a week.

### Proliferation Assay

Cell proliferation was assessed by modified MTT assay according to the manufacturer's recommendations (CellTiter 96® Non-Radioactive Cell Proliferation Assay, Promega). Briefly, cells were seeded at a concentration of 5,000 cells/well in a 96-well plate and allowed to attach for 4 h. Cells were then exposed to a range of concentrations from 0.156 to 80 μM of WD for 48 h. DMSO (0.8%) served as vehicle and control. After adding reagents according to the manufacturer's recommendations, absorbance was recorded at 570 nm using an Epoch Microplate Spectrophotometer (Biotek, Vermont, USA). The concentration of drugs that resulted in 50% of cell death (IC_50_) was determined from dose-response curve by using PRISM 7.0 (GraphPad Software, San Diego, CA, USA). Experiments were repeated three times, and data represented as the mean of quadruplicate wells ± SEM.

### Irradiation

Irradiation was performed using a cabinet X-ray machine (X-RAD 320, Precision X-Ray Inc.) at 320 kVp and 12.5 mA with a 2 mm Al filter. The source-to-axis distance was 42 cm. The beam was calibrated using a UNIDOS E PTW T10010 electrometer and TN30013 ionization chamber, with measurement done in air, for a 15 cm × 15 cm field size. The dose rate was 3 Gy/min.

### Clonogenic Assay

Clonogenic assay was performed as previously described (12). Briefly, cells were seeded in six well plates (100, 200, 1,000, and 2,000 cells/well for DMSO-treated cells irradiated at 0, 2, 4, and 6 Gy, respectively, and 200, 400, 4,000, and 8,000 cells/well for WFA- or WD-treated cells irradiated at 0, 2, 4, and 6 Gy, respectively), allowed to attach for 4 h and then exposed to WFA, WD or DMSO at a concentration of 0.7 μM, 1 h before irradiation. They were then irradiated at 0, 2, 4, or 6 Gy. The medium was removed immediately after irradiation and replaced by a drug-free medium, then changed once a week. Cells were fixed with 6% of glutaraldehyde and stained with 0.5% of crystal violet approximatively after 14 days. Colonies >50 cells were counted manually using a binocular loupe. Experiments were repeated three times, and data represented the mean of duplicate wells ± SEM.

### Alkaline Comet Assay

SKOV3 cells were seeded in T-25 flasks (200,000 cells/mL) and exposed to DMSO or WD when reached 70% confluence at a concentration of 0.7 μM 1 h before being irradiated at 2 Gy. The medium was removed right after irradiation and replaced by a drug-free medium. Alkaline comet assay was then performed 1 or 24 h after irradiation as previously described (13). Briefly, cells were harvested and resuspended in PBS at a density of 20,000 cells/mL. Successively, 400 μL sample of this cell suspension was mixed with 1.2 mL of 1% low melting agarose, and 1 mL of this mixture was disposed on microscope slides previously coated with 1% low melting agarose. Slides were treated with alkaline lysis solution (1.2 M NaCl, 100 mM Na_2_EDTA, 0.1% sodium lauryl sarcosinate, 0.26 M NaOH) for 20 h at 4°C. Slides were successively washed three times in 0.03 M NaOH, 2 mM Na_2_EDTA (alkaline buffer) for 20 min each, followed by electrophoresis in alkaline buffer for 25 min at 0.6 V/cm. Then, slides were washed and neutralized in distilled water and finally stained with sytox green (1 mM) immediately before imaging. Olive tail moment (defined as the percent DNA in the tail multiplied by the distance between the means of the head and tail distributions) was analyzed and quantified using CaspLab software.

### Immunofluorescence

SKOV3 cells were seeded on collagen-coated coverslip at a density of 10,000 cells/well. Cells were exposed to DMSO or WD at a concentration of 0.7 μM 1 h before being irradiated at 2 Gy. The medium was removed right after irradiation and replaced by a drug-free medium. Cells were successively fixed for 15 min in 4% PFA, and permeabilized with 0.1% Triton X-100 for 5 min at room temperature at 1, 6, and 24 h post-irradiation for γH2Ax/53BP1 foci formation assay and 24 h only for mitotic catastrophe assay. After permeabilization, cells were blocked for 30 min with 1% BSA at room temperature before being incubated for 1 h with antibodies diluted in blocking buffer and raised against 53BP1 (Abcam, ab21083, 1:400), phospho-histone H2AX (Merck Millipore, 05-636, 1:800), α-tubulin (Santa Cruz Biotechnology, sc-5286, 1:150), or pericentrin (Abcam, ab4448, 1:4,000). After thorough washes with PBS, cells were incubated for 30 min in the dark with Cy™ 3-conjugated AffiniPure Goat Anti-Mouse IgG (Jackson ImmunoResearch, 115-165-062, 1:400) and Alexa Fluor® 647-conjugate AffiniPure Goat Anti-Rabbit IgG (Jackson ImmunoResearch, 111-605-045, 1:200). The nuclei were counterstained with 4′,6-diamidino-2-phenylindole (DAPI) for 5 min. Coverslip were mounted on the slides with a drop of mounting medium and imaged using an Epifluorescence microscope (Zeiss Axio Imager M2).

### Immunoblotting

SKOV3 cells were seeded in T-25 flasks (200,000 cells/mL) and exposed to DMSO or WD when they reached 70% confluence at a concentration of 0.7 μM 1 h before being irradiated at 2 Gy. The medium was removed right after irradiation and replaced by a drug-free medium. Cells were directly lyzed using the radioimmunoprecipitation assay (RIPA) buffer at 1, 6, and 24 h post-irradiation. Proteins were quantified in 96-well plates by using the Pierce BCA Protein Assay kit (Thermo Fisher Scientific) following manufacturer's recommendations. Five micrograms of total proteins were then eluted by adding Laemmli buffer, and heated at 95°C for 10 min. Samples were size-separated by electrophoresis on 4–20% gradient acrylamide Tris-HCl Protein precast gels (Biorad) at 90 V for 2 h in 25 mM Tris, 192 mM glycine, 0.1% SDS buffer, and transferred to PVDF membranes (Invitrogen) at 300 mA for 90 min in 25 mM Tris, 192 mM glycine, 0.1% SDS with 20% ethanol. Membranes were blocked with PBST (PBS plus 0.1% Tween-20) containing 5% non-fat milk at room temperature for 60 min. Blots were then incubated (4°C, overnight) with primary antibodies against ATM (Cell signaling Technology, 2873, 1:1,000), pATM (phospho S1981) (Abcam, ab81292, 1:50,000), DNA-PKcs (Santa Cruz Biotechnology, sc-5282, 1:200), pDNA-PKcs (phospho S2056) (Abcam, ab18192, 1:1,000), GAPDH (Santa Cruz Biotechnology, sc-365062, 1:200), Rad51 (Abcam, ab63801, 1:100), and XRCC4 (Santa Cruz Biotechnology, sc-271087, 1:100). After five washes (5 min/each) with PBST, blots were incubated with a horse radish peroxidase (HRP) conjugated goat secondary antibody against rabbit IgG (Jackson ImmunoResearch, 111-035-003, 1:5,000) or mouse IgG (Sigma, A 4416, 1:10,000) at room temperature for 1 h. After washing five times with PBST (5 min/each), blots were developed with Clarity™ Western ECL substrate and imaged using a ChemiDoc™ XRS+ system (BioRad).

### Statistical Analysis

Data are expressed as the mean ± SEM. Statistical analysis were performed by non-parametric Wilcoxon signed-rank test or Wilcoxon–Mann–Whitney test. Welch's *t*-test was used when sample size was too small for the non-parametric tests. *P*-value (*p*) < 0.05 (two-sided) was considered statistically significant for all of the statistical calculations. All statistical analyses and graphs were performed with PRISM 7.0 (GraphPad Software, San Diego, CA, USA).

## Results

### WD Inhibited Cell Proliferation of Various Cancer Cell Types

Cell proliferation in prostate (22Rv1, DU145), lung (A549), intestinal (Caco2), breast (MCF7), and ovarian (SKOV3) cancer cells exposed to different concentrations of WD for 48 h was determined by modified MTT assay. Cell viability decreased significantly in all cell lines after WD exposure in a concentration-dependent manner (Supplementary Figure 5). To estimate the IC_50_ concentration, we normalized cell viability from 100% in DMSO and performed a nonlinear regression using dose response curve fitting [log(inhibition) vs. normalized response (variable slope)] in GraphPad Prism. Results showed that WD had an IC_50_ < 3 μM in all cell lines, with Caco-2 being the most sensitive (IC_50_ = 0.63 μM) and SKOV3 the most resistant (IC_50_ = 2.93 μM) among the tested cell lines (Supplementary Table 1). For further molecular studies and protein expression profiling, a sub-cytotoxic concentration of 0.7 μM was selected, which is therapeutically relevant and effective in cancer xenograft studies *in vivo* (14, 15).

### WD Showed a Higher Radiosensitizing Effect Than WFA in Several Cancer Cell Lines

**Figure 2:**
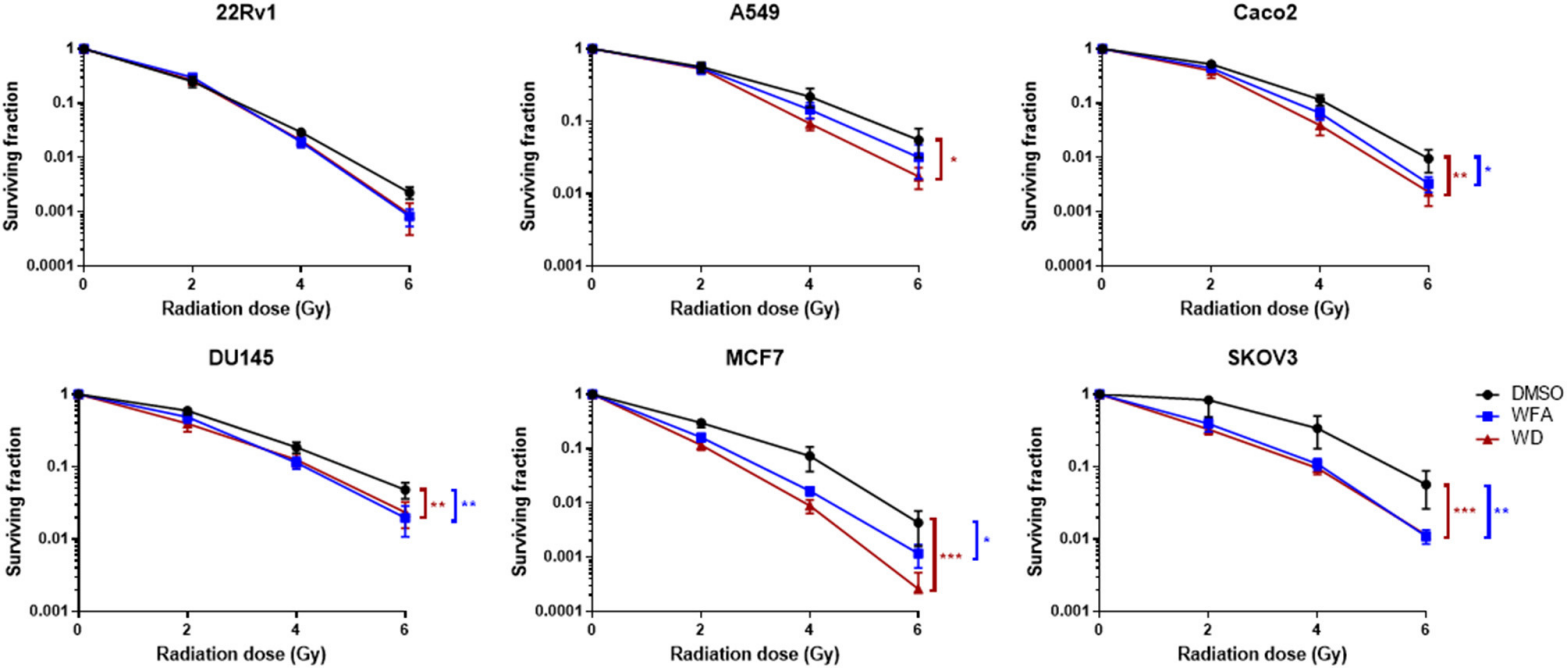
WD sensitized several cancer cell lines to X-Ray irradiation. Cells in log phase were pretreated with 0.7 μM of WFA, WD or vehicle (DMSO) for 1 h and then irradiated at indicated doses. Data points represent colonies >50 cells as the mean ± SEM from three independent experiments with at least two replicates each. Significantly different values as determined by Wilcoxon signed-rank test (*p < 0.05, **p < 0.01, ***p < 0.001).

A colony formation assay was then performed to evaluate the radiosensitizing effect of WFA and WD. As shown in Figure 2, pre-irradiation treatment of human cancer cells with WD for 1 h significantly decreased the surviving fraction after X-ray irradiation for Caco-2 (*p* = 0.021), DU145 (*p* = 0.0025), MCF7 (*p* = 0.0002), A549 (*p* = 0.0159), and SKOV3 (*p* = 0.001) compared to cells only exposed to DMSO. Interestingly, the sensitizing enhancement ratio (SER_0.1_) of WFA and WD calculated at the D10 value was superior for WD than WFA for all cell lines, suggesting that WD has a higher radiosensitizing effect (Table 1). In order to determine the mechanisms by which WD achieved its radiosensitizing effect, SKOV3 cells, which displayed the highest radiosensitivity to WD + irradiation (SER_0.1_ = 2.22, Table 1) were selected.

**Table 1:** Sensitizing enhancement ratio (SER_0.1_) of WFA and WD drugs was calculated at the D10 value as the radiation dose needed for radiation alone divided by the dose needed for WFA/WD plus radiation at a survival fraction of 10%.

**Cell line**	**SER**_****0.1****_	
	**WFA**	**WD**
22Rv1	0.92	0.99
A549	1.12	1.21
Caco2	1.17	1.29
DU145	1.21	1.37
MCF7	1.49	1.75
SKOV3	1.97	2.22

### WD Induced a Persistence of DNA Damages, and Increased the Expression of γH2AX and 53BP1 Foci in Irradiated Cells

**Figure 3:**
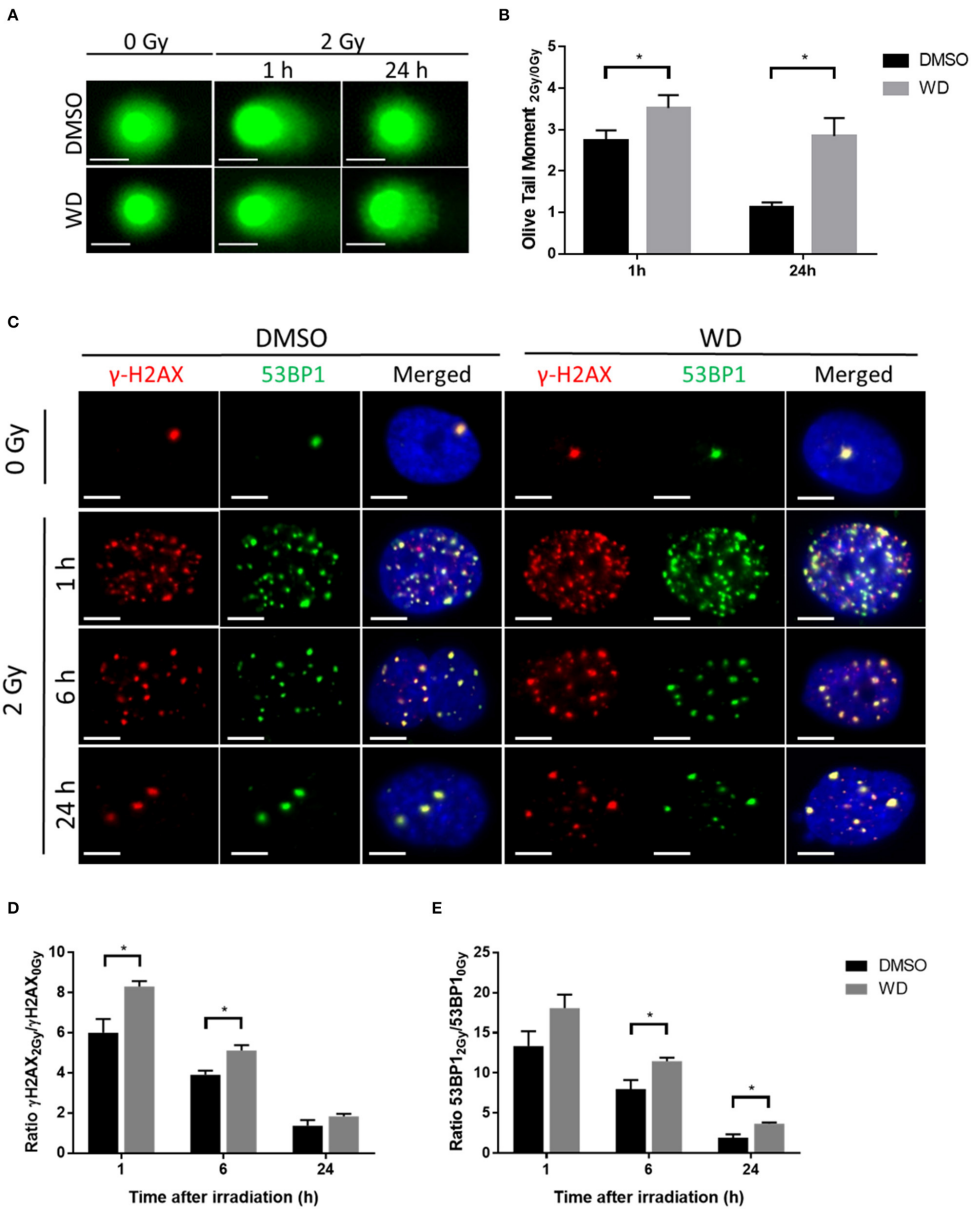
WD prolonged radiation-induced DNA damages in SKOV3 cells. **(A)** Representative images of alkaline comet assay of SKOV3 cells treated with vehicle (DMSO) or WD (0.7 μM) for 1 h and then irradiated at 2 Gy followed by 1 and 24 h incubation. Scale bar, 25 μm. **(B)** Average olive tail moment of at least 50 cells from two independent experiments. Data points represent the mean ± SEM. *Significantly different values as determined by Wilcoxon–Mann–Whitney test (*p* < 0.05). **(C)** Immunofluorescence showing γH2AX (red) and 53BP1 (green) foci formation in SKOV3 cells pre-treated with vehicle (DMSO) or WD (0.7 μM) for 1 h and then irradiated at 2 Gy followed by 1, 6, and 24 h incubation. Nuclei were counterstained with DAPI (blue). Scale bar, 10 μm. **(D)** Ratio of radiation-induced γH2AX and **(E)** 53BP1 foci normalized to sham-irradiated cells, exposed to DMSO or WD. Data points represent the mean ± SEM of at least 60 nuclei from three independent experiments. Significantly different values as determined by Wilcoxon–Mann–Whitney test (*p < 0.05).

Promotion of radiation-induced DNA damages and inhibition of DNA double-strand breaks (DSBs) repair by WD was first assessed in SKOV3 cells. Alkaline comet assay was performed to ascertain the combination of radiation-induced DNA single-strand breaks, double-strand breaks and alkali-labile sites after 1 h pre-treatment with WD. Results showed that 2 Gy-irradiation induced formation of comet tail at 1 h which returned to baseline level at 24 h (Figure 3A). Interestingly, olive tail moment normalized with non-irradiated cells was significantly higher at 1 and 24 h (*p* = 0.0086 and *p* < 0.0001, respectively) post-irradiation in cells exposed to WD compared to those exposed to vehicle (Figure 3B). As DSBs are the most lethal radiation-induced damages, this type of lesion was further investigated and the formation of γH2AX and 53BP1 foci, two markers of DSBs, was monitored in SKOV3 cells irradiated with X-rays with or without WD pre-treatment. The number of co-localized γH2AX and 53BP1 foci highly increased 1 h after 2 Gy-irradiation compared to sham-irradiated cells. At 6 h post-irradiation, the number of foci started to decrease to return to normal level at 24 h (Figure 3C and Supplementary Figures 6A,B). However, SKOV3 cells pre-treated with WD presented more γH2AX foci at 1 and 6 h post-irradiation (*p* = 0.0095 and *p* = 0.019, respectively) and more 53BP1 foci at 6 and 24 h post-irradiation (*p* = 0.026 and *p* = 0.041, respectively) than sham-irradiated cells compared to DMSO-treated cells (Figures 3D,E). Together, these data suggest that WD induced the persistence of DNA damages and especially DSBs.

### WD Down-Regulated DNA Damage Repair Proteins in Irradiated SKOV3 Cells

**Figure 4:**
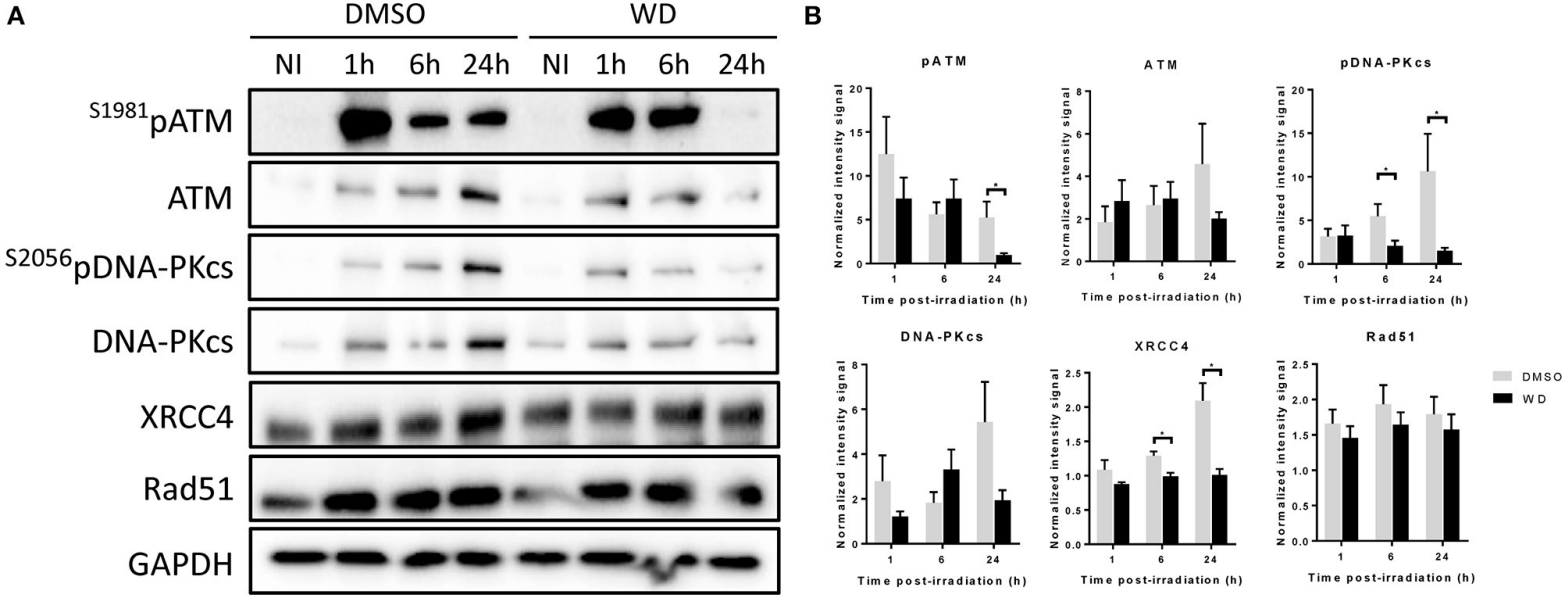
WD inhibited DNA repair proteins in irradiated SKOV3 cells. **(A)** Western blot is showing the expression level of ATM, ^S1981^pATM, DNA-PKcs, ^S2056^pDNA-PKcs, XRCC4, and RAD51 proteins in SKOV3 cells exposed to vehicle (DMSO) or WD (0.7 μM) for 1 h prior to sham- or 2 Gy-irradiation and incubated for 1, 6, or 24 h. NI, Non-Irradiated samples. **(B)** Bands intensities were quantified using ImageJ and normalized with GAPDH. Data points represent the mean ± SEM from three independent experiments. Significantly different values as determined by Wilcoxon–Mann–Whitney test (*p < 0.05).

In order to further investigate how WD prolonged DNA damages, the expression of proteins involved in DNA damage repair was assessed by western blot. SKOV3 cells were pretreated with WD for 1 h before sham or 2 Gy-irradiation. WD was immediately replaced after irradiation by drug-free medium for 1, 6, and 24 h. ATM and DNA-PK kinases, and their phosphorylated forms were overexpressed from 1 h after irradiation and maintained at a high expression level for at least 24 h in DMSO-treated cells (Figure 4). However, in WD-treated cells, their expression level decreased rapidly and almost returned to baseline level after 24 h compared to sham-irradiated samples. XRCC4 protein was overexpressed after irradiation and maintained high level up to 24 h in DMSO-treated cells whereas its expression stayed stable post-irradiation compared to sham-irradiated cells in WD-treated cells. Conversely, Rad51 expression level was identically increased after irradiation between DMSO- and WD-treated cells.

### WD Induced Mitotic Catastrophe (MC) in Irradiated SKOV3 Cells

**Figure 5:**
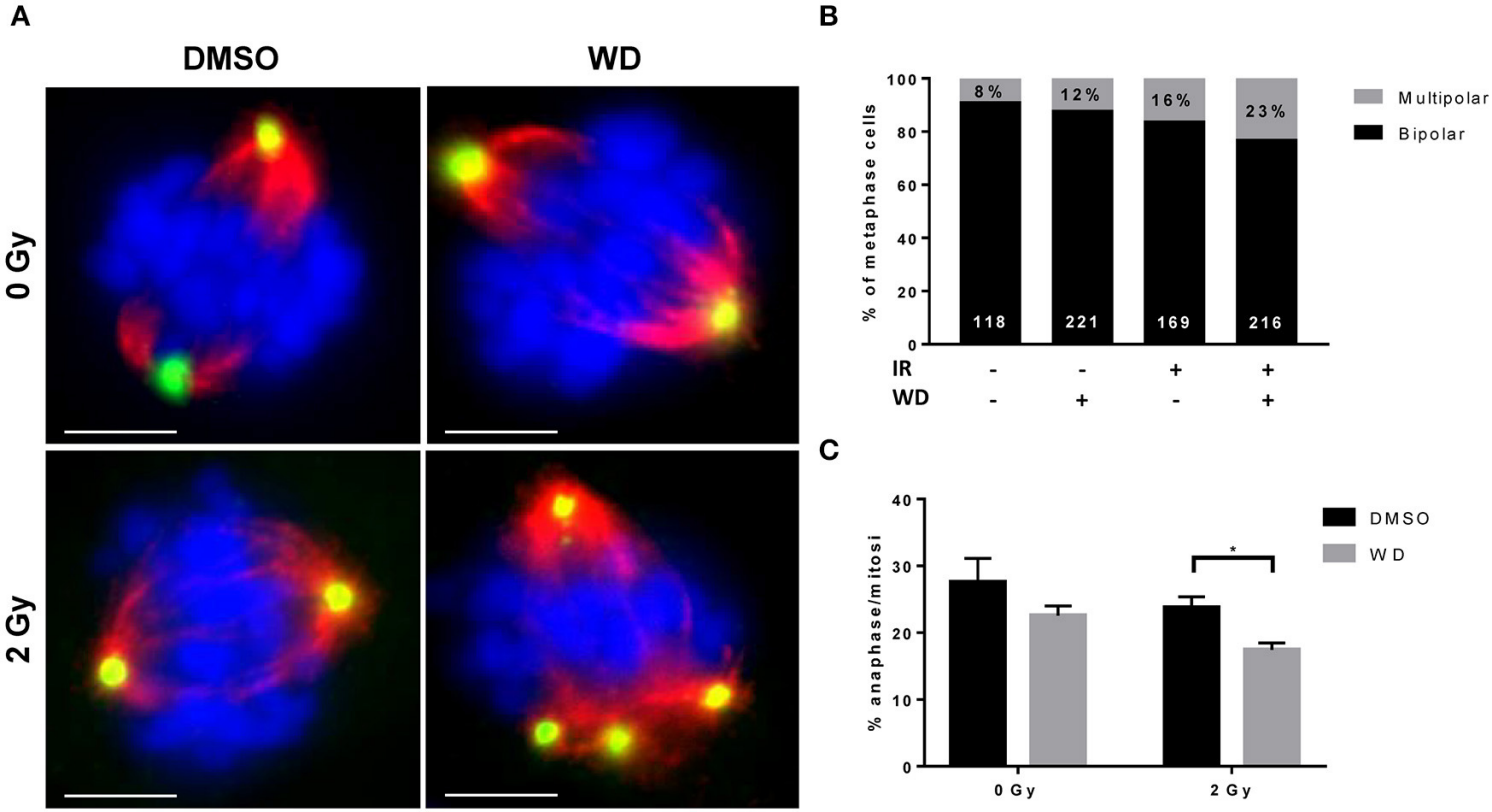
WD induced MC in irradiated SKOV3 cells. **(A)** Representative images of immunostaining of mitotic spindle with anti α-tubulin (red) and pericentrin (green) in SKOV3 cells exposed to vehicle (DMSO) or WD (0.7 μM) for 1 h prior to sham- or 2 Gy-irradiation and incubated for 24 h. Nuclei were counterstained with DAPI (blue). Scale bar, 15 μm. Quantification of mitotic catastrophe as **(B)** the percentage of multipolar metaphases (in black on each bar chart, the number of metaphases scored is reported in white at the bottom of each bar chart) and **(C)** the percentage of cells in anaphase among cells in mitosis (from at least 200 mitotic events from three independent experiments). Significantly different values as determined by Welch's *t*-test (*p < 0.05).

Recent evidences showed that the combined loss of ATM and p53 promoted cellular death due to MC after DSBs (16, 17). SKOV3 is a p53-deficient cell line (18) and recent study showed that cisplatin-induced DNA damage triggered MC (19). Since our results also demonstrated a loss of ATM expression after irradiation when combined with WD exposure, we anticipated that WD radiosensitizing effect could promote cellular death by MC. Immunofluorescence assays for α-tubulin and pericentrin stains showed that control cells displayed normal bipolar mitotic spindles, whereas WD-treated cells exhibited an abnormal mitotic spindle with large defects in chromosomal alignment, such as the existence of multi-polar spindle morphology 24 h after 2 Gy-irradiation (Figure 5A). The proportion of multipolar metaphases in WD-treated cells exposed to irradiation increased compared to non-exposed cells and WD-exposed or irradiated cells alone (Figure 5B). In addition, cells displaying aberrant spindles did not progress through anaphase, and the percentage of cells in anaphase significantly decreased for cells exposed to WD and irradiation (*p* = 0.0439) (Figure 5C).

## Discussion

Although the radiosensitizing effect of WFA has already been demonstrated, this study confirmed the previous observation and also showed, for the first time, the higher radiosensitizing effect of WD. This radiosensitizing effect was obtained by performing a 1 h WD pretreatment at a pre-determined concentration of 0.7 μM. However, since the effect of WD toxicity on cell proliferation varied for each cell line, different concentrations should be tested to optimize this radiosensitizing effect. Our results suggest that WD prolonged radiation-induced DNA damages by inhibiting DNA damage repair. DNA-PKcs and ATM are the two main kinases involved in response to DSBs (20) and both were shown to be inhibited by the presence of WD after irradiation. DNA-PKcs is usually considered as a core regulator and biomarker of non-homologous end joining pathway (NHEJ) repair pathway while ATM as the initiator of DNA repair via homologous recombination (HR) (21, 22). However, recent studies demonstrated that this separation is not as simple and that the two proteins play an important role in both pathways (23, 24). Indeed, while HR is elevated in DNA-PKcs null cells, DNA-PKcs kinase inhibitors has been reported to suppress HR in normal cells, highlighting possible role of DNA-PK in HR pathway fate (25). Moreover, ATM levels are regulated by DNA-PKcs (26) and ATM also phosphorylates DNA-PKcs after irradiation (27), suggesting that these two proteins cooperate to regulate DNA DSBs repair by NHEJ and HR. Therefore, effector proteins specifically involved in NHEJ and HR pathways such as XRCC4 and Rad51, respectively, were further investigated (28). Results showed that the expression level of XRCC4 and, interestingly, ^S2056^pDNA-PKcs, decreased in WD pre-treated irradiated cells. Phosphorylation of DNA-PKcs is required to induce conformational changes at broken ends and allows access to XRCC4 complex at these sites to ligate both loose ends (29). Therefore, both WD-induced XRCC4 and ^S2056^pDNA-PKcs inhibition could result in much reduced fidelity and efficiency of NHEJ. Rad51 expression remained unchanged when irradiated cells were pre-treated with DMSO or WD, suggesting that the compound did not inhibit HR pathway. However, in addition to this immunoblot analysis, supplementary investigations would be necessary to certainly confirm NHEJ pathway inhibition.

This delay in radiation-induced DNA damage repair lead the cells to MC. This result is consistent with previous studies which reported that mutant p53 and inhibition of G2 checkpoint proteins such as ATM, promote radiation-induced MC (30–32). A recent study showed that WD induces apoptosis in p53-wild type cells through Bax/Bak dependent pathway, but that Bak can compensate against loss of Bax in p53-null cells and thus still induces apoptosis (33). However, we did not observe an increase of apoptosis level in SKOV3 p53-null cells when analyzed by TUNEL assay (data not shown). This may be explained by the low dose (0.7 μM) and short incubation time (1 h) that we voluntary selected in this study to induce a radiosensitizing effect while limiting the cytotoxic effect of WD. In addition, since the combined loss of ATM and p53 inhibit cell cycle checkpoints (16), we did not investigate cell cycle in SKOV3 cells. However, WD has been shown to induce G2/M phase arrest in pancreatic adenocarcinoma cells (34). Thus, additional investigations should be performed to assess if cell cycle arrest could play a role in WD radiosensitizing effect, in particular in p53-wild type cells. Indeed, because of their highest SER_0.1_, mechanistic analyses in this study focused on p53-null SKOV3 cells, however, WD also radiosensitized other cancer cell lines to a lesser degree, including p53-functional cell lines (i.e., A549 and MCF7). This suggests that the radiosensitizing effect of WD is independent of p53 status and the fate of radiation-induced cell death in p53-effective cells would mainly depend on WD-induced ATM (or DNA-PKcs) inhibition. WD has been previously shown to increase JNK and p38MAPK phosphorylation (35), two pathways strongly responsive to ionizing radiation and that influence tumor cell radiosensitivity because of their activity associated with radiation-induced DNA damage response (36). Radiation-induced JNK activation is dependent on ATM and its activation activates caspases and regulates proteins implicated in apoptosis regulation, including p53 (36, 37), while MAPKK-p38γ cascade is required for radiation–induced G2 arrest (38). These mechanisms could explain the radiosensitizing effect of WD that we observed in p53 wild-type cell lines. In addition, JNK and p38MAPK activation enhances the ceramide accumulation, an important messenger for radiation response that triggers radiation-induced apoptosis (39–42). The effect of WD on the ceramide production and MAPKK pathways activation upon irradiation needs to be investigated further to assess if it plays a role on the radiosensitizing effect of WD, independently, or not, of p53. Further studies involving additional cell lines, with different p53 status, and *in vivo* experiments would be required to surely affirm radiosensitizing effect of WD and its cellular effect.

After WFA, WD highlighted the potential of withanolides to act as promising sources of radiosensitizers. Several studies investigated the relationship between the structure and the diverse bioactivities reported for withanolides to better understand their mechanism of action and modify the withanolide scaffold accordingly to enhance its effect (43–46). Although WD-treated cells displayed a lower expression level of DNA damage repair proteins after irradiation than non-treated cells (maximal at 24 h), this inhibition is not complete. Thus, additional studies are required to identify the key structural components of withanolides responsible for this inhibition in order to optimize their radiosensitizing effect.

## Data Availability Statement

All datasets generated and analyzed for this study are included in the article/Supplementary Material.

## Author Contributions

JL and FZ designed the research. JL, TC, LM, and EW conducted the experiments. JL, TC, and LM analyzed the data. JL drafted the manuscript. J-LV, MC, AG, and FZ revised the manuscript. All authors read and approved the final manuscript.

### Conflict of Interest

The authors declare that the research was conducted in the absence of any commercial or financial relationships that could be construed as a potential conflict of interest.

## Abbreviations

DSBs: double-strand breaks
HR: homologous recombination
MC: mitotic catastrophe
NHEJ: non-homologous end joining
RT: radiation treatment
WD: withanolide D
WFA: withaferin A.
