# Supplementary material for: Withanolide D Enhances Radiosensitivity of Human Cancer Cells by Inhibiting DNA Damage Non-homologous End Joining Repair Pathway
Source: Front Oncol. 2020 Jan 8;9:1468. doi: 10.3389/fonc.2019.01468 (PMC6960174; doi:10.3389/fonc.2019.01468)
Supplement: Supplementary file 1 [file Data_Sheet_1.PDF]

Supplementary Material

Supplementary Figures

NPC, UA

HPLC Report

Agilent Technologies

```
Sample Name:          Withaferin A
Data File Name:       C:\CHEM32\1\DATA\KITHSIRI WIJERATNE\MC000089.D
Method Name:          C:\CHEM32\1\METHODS\KITHSIRI.M
data acquired by:   Kithsiri Wijeratne
on:                 8/29/2019
location:           Vial 1
```

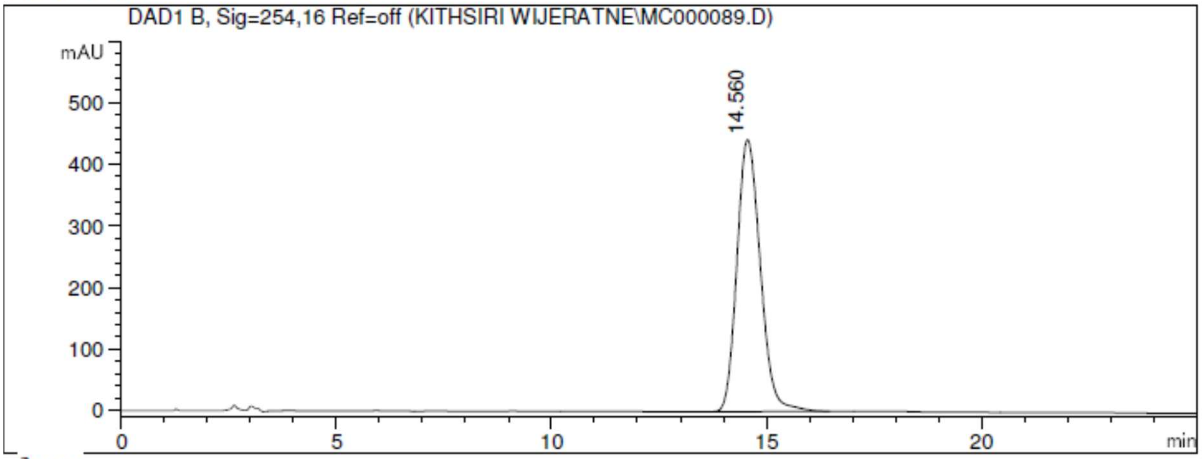

Area

#	Meas. R	Height	Height %	Area	Area %
1	14.560	441.930	100.000	16713.143	100.000

Supplementary Figure 1. HPLC Analysis of WFA confirming its purity (>98%). HPLC analysis was performed on an Agilent 1100 series HPLC system (GenTech) equipped with G1379A degasser, G13111A Quart Pump, and G1315B diode array detector and Agilent ChemStation for LC & LCMS systems using Kromasil C_{18} 5.0 µm (250 x 4.6 mm) column (Supelco Inc). Injections (20.0 µL) were made with G1329A Auto sampler. Mobile phase consisted of CH_3OH/H_2O (7:3, isocratic) with a flow rate of 0.8 mL/min. Chromatograms were detected at 254 nm.

NPC, UA

HPLC Report

Agilent Technologies

Sample Name: Withanolide D
Data File Name: C:\CHEM32\1\DATA\KITHSIRI WIJERATNE\MC000091.D
Method Name: C:\CHEM32\1\METHODS\KITHSIRI.M
data acquired by: Kithsiri Wijeratne
on: 8/29/2019
location: Vial 1

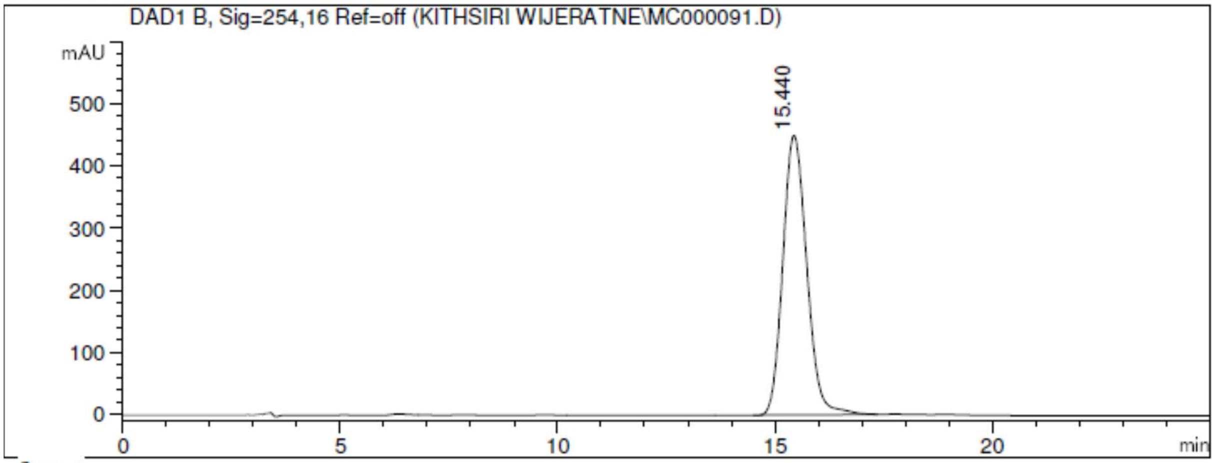

Area

#	Meas. R	Height	Height %	Area	Area %
1	15.440	450.025	100.000	17248.277	100.000

Supplementary Figure 2. HPLC Analysis of WD confirming its purity (>98%). HPLC analysis was performed on an Agilent 1100 series HPLC system (GenTech) equipped with G1379A degasser, G13111A Quart Pump, and G1315B diode array detector and Agilent ChemStation for LC & LCMS systems using Kromasil C_{18} 5.0 µm (250 x 4.6 mm) column (Supelco Inc). Injections (20.0 µL) were made with G1329A Auto sampler. Mobile phase consisted of CH_3OH/H_2O (7:3, isocratic) with a flow rate of 0.8 mL/min. Chromatograms were detected at 254 nm.

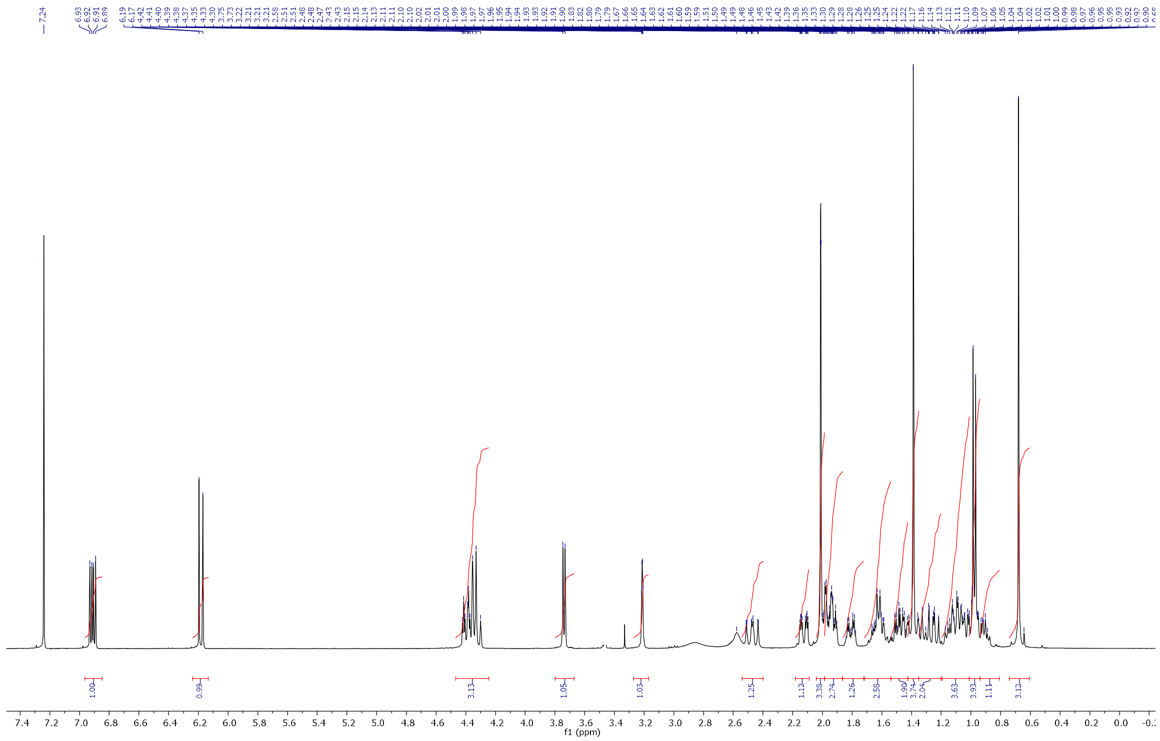

Supplementary Figure 3. ^{1}H NMR (400 MHz) spectrum of WFA in $CDCl_3$ determined using a Bruker Avance III 400 spectrometer. The signal (δ 7.24) for residual $CHCl_3$ was used as the internal reference.

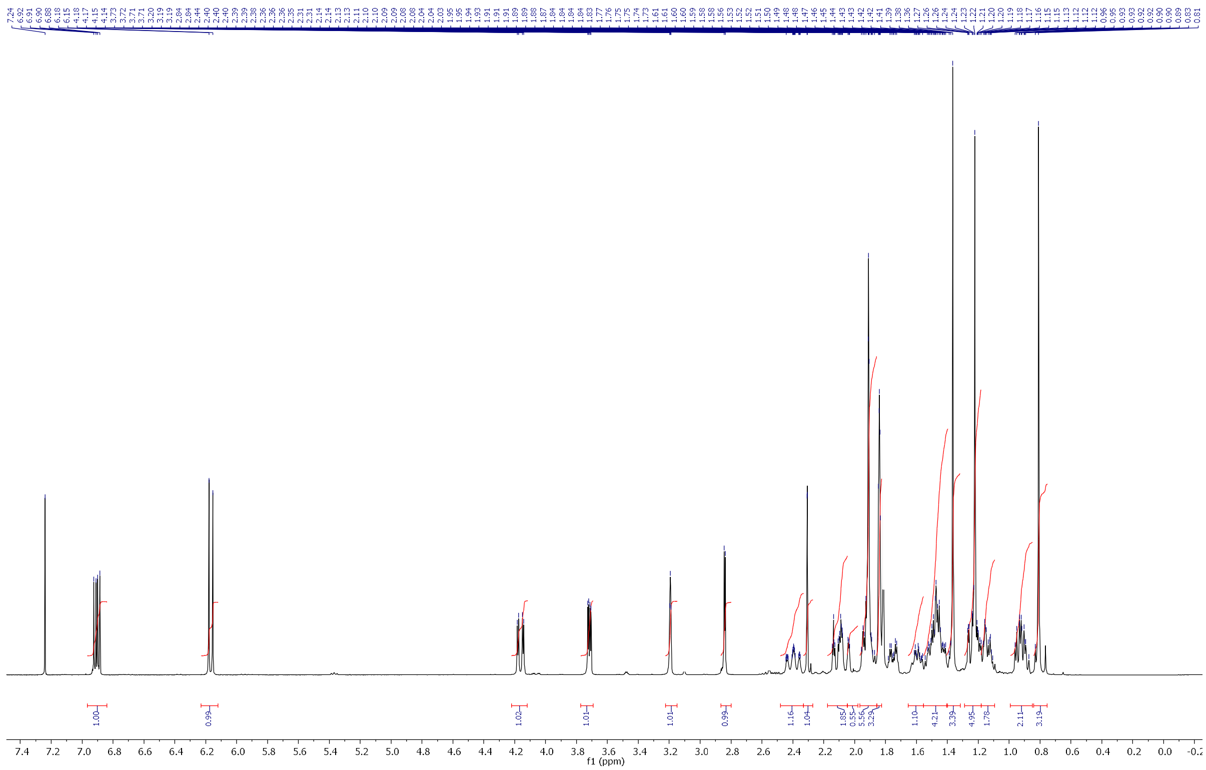

Supplementary Figure 4. ^{1}H NMR (400 MHz) spectrum of WD in $CDCl_3$ determined using a Bruker Avance III 400 spectrometer. The signal (δ 7.24) for residual $CHCl_3$ was used as the internal reference.

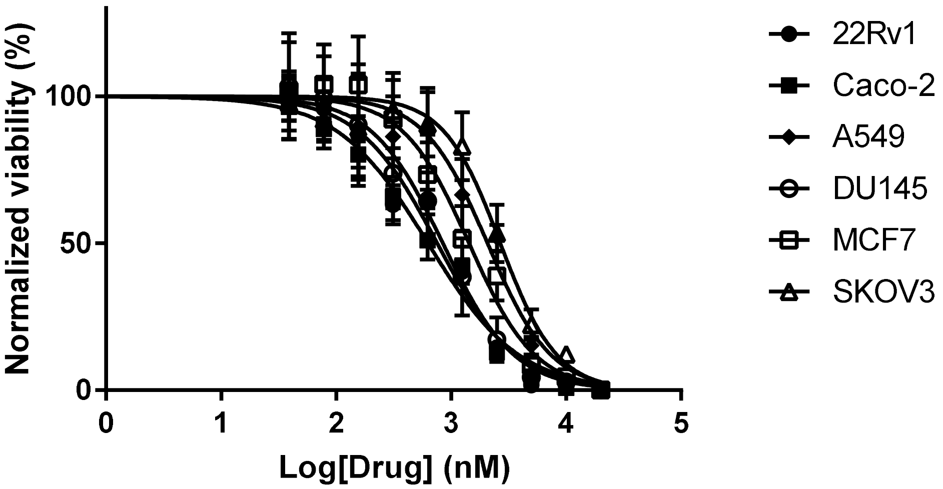

Supplementary Figure 5. Effect of WD treatment on several cancer cell lines viability. (A) Five thousand cells were seeded in a 96 well plate and exposed to a large range of concentrations (0.156 to 80 μM) of WD for 48 h. Cell viability was assessed by a modified MTT assay (CellTiter 96® Non-Radioactive Cell Proliferation Assay). Absorbance (570 nm) values were normalized from 100% in DMSO control and analyzed using a nonlinear regression using dose response curve fitting (log (inhibition) vs normalized response (variable slope)). Data points represent the mean ± SEM from three independent experiments with at least four replicates each.

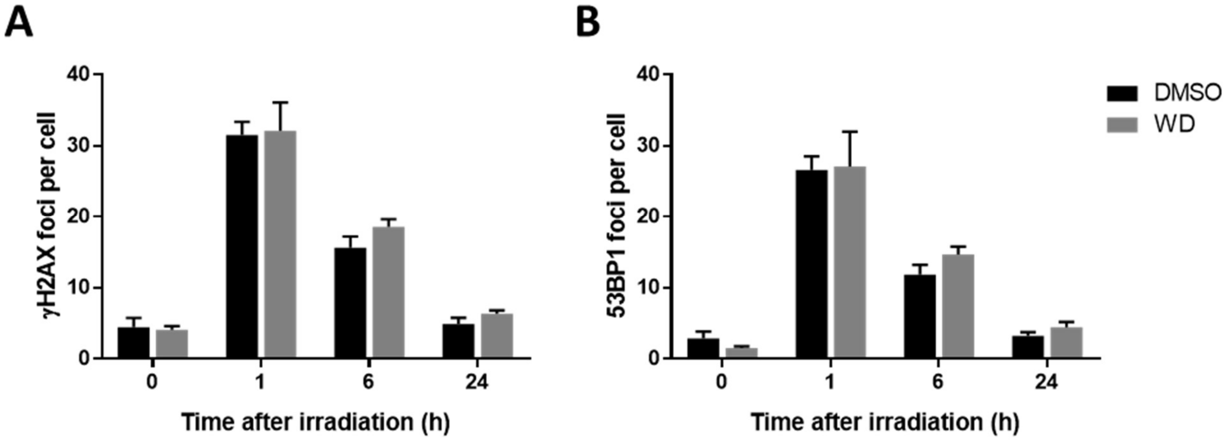

Supplementary Figure 6. (A) Radiation-induced γH2AX and **(B)** 53BP1 foci in SKOV3, exposed to 0.7μM WD for 1 h and then irradiated at 2Gy. Data points represent the mean ± SEM of at least 60 nuclei from 3 independent experiments.

.

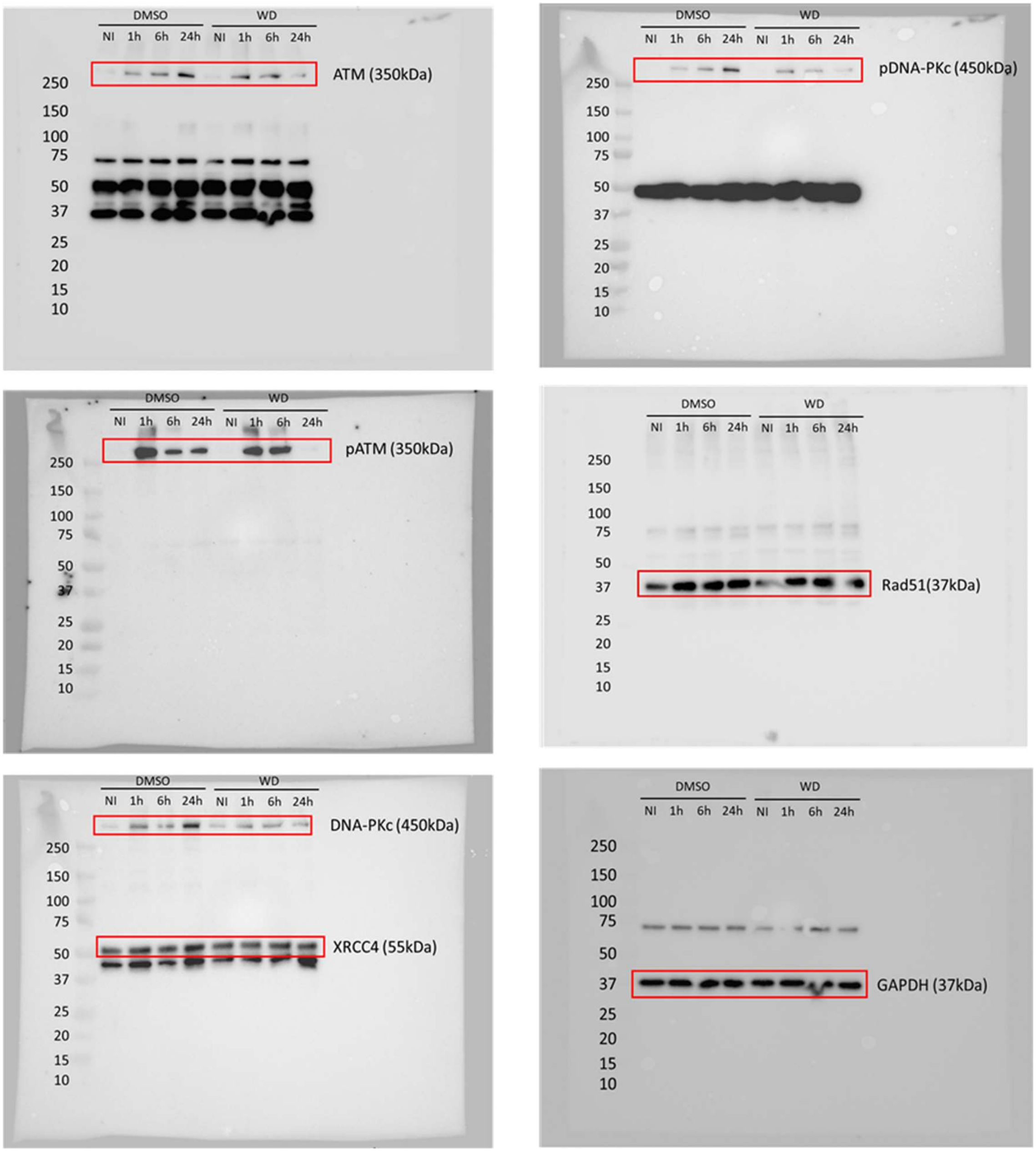

Supplementary Figure 7. Uncropped western blot images. Red rectangles correspond to the cropped sections visible in Figure 4.

Supplementary Table

Supplementary Table 1. IC_{50} values (μM) at 48 h exposure time were determined for WD.

**Cell line**	**IC_{50}**
22Rv1	0.76
A549	2.34
Caco2	0.63
DU145	0.91
MCF7	1.53
SKOV3	2.93
